# Update on the Management of Parkinson's Disease for General Neurologists

**DOI:** 10.1155/2020/9131474

**Published:** 2020-03-26

**Authors:** Zvezdan Pirtošek, Ovidiu Bajenaru, Norbert Kovács, Ivan Milanov, Maja Relja, Matej Skorvanek

**Affiliations:** ^1^Department of Neurology, University Medical Center & Chair of Neurology, Medical Faculty University of Ljubljana, Ljubljana, Slovenia; ^2^Department of Clinical Neurosciences, University of Medicine and Pharmacy “Carol Davila”, Bucharest, Romania; ^3^Department of Neurology Medical School University of Pecs, Pecs, Hungary; ^4^Department of Neurology, Multiprofile University Hospital for Active Treatment in Neurology and Psychiatry “St. Naum”, Sofia, Bulgaria; ^5^Movement Disorders Center, Department of Neurology, School of Medicine, University Hospital Center Zagreb, Zagreb, Croatia; ^6^Department of Neurology. Medical Faculty, P. J. Safarik University and Department of Neurology University Hospital of L. Pasteur, Kosice, Slovakia

## Abstract

Management of Parkinson's disease (PD) is complicated due to its progressive nature, the individual patient heterogeneity, and the wide range of signs, symptoms, and daily activities that are increasingly affected over its course. The last 10–15 years have seen great progress in the identification, evaluation, and management of PD, particularly in the advanced stages. Highly specialized information can be found in the scientific literature, but updates do not always reach general neurologists in a practical and useful way, potentially creating gaps in knowledge of PD between them and neurologists subspecialized in movement disorders, resulting in several unmet patient needs. However, general neurologists remain instrumental in diagnosis and routine management of PD. This review provides updated practical information to identify problems and resolve common issues, particularly when the advanced stage is suspected. Some tips are provided for efficient communication with the members of a healthcare team specialized in movement disorders, in order to find support at any stage of the disease in a given patient, and especially for a well-timed decision on referral.

## 1. Introduction

Parkinson's disease (PD) is a disorder with increasing prevalence worldwide, and the second most common neurodegenerative disorder, surpassed only by Alzheimer's disease [1, 2]. Management remains complex over the course of PD due to its progressive nature, individual patient heterogeneity, and wide range of signs, symptoms, and increasingly affected daily functions. However, the last 10–15 years have seen great progress in the identification, evaluation, and management of the disease, particularly in the advanced stages [3]. This information does not always reach general neurologists in a practical and useful way, potentially creating gaps in knowledge of PD between general neurologists (GNs) and specialists in movement disorders (MD), resulting in several unmet patient needs. Nonetheless, GNs remain instrumental in diagnosis and routine management in earlier stages of the disease. Their ability to identify problems, resolve common issues, and identify signs of the emerging advanced stage of PD is paramount for appropriate management [4], efficient communication with members of the MD team, and also a well-timed decision on referral.

This article therefore aims to provide a practical overview of the most up-to-date information from the recent literature, as well as relevant issues in the management of PD, in order to support GNs in decision-making and communication with members of the MD healthcare team.

## 2. General Characteristics of Parkinson's Disease

### 2.1. Clinical Manifestations and Diagnosis

Clinical manifestations of PD may be classified into two groups: motor symptoms (MS) and nonmotor symptoms (NMS), and they evolve through three main stages: (1) a preclinical stage, (2) a premotor stage (with only some NMS present), and (3) a motor stage with MS. The GN should suspect PD in people presenting with resting tremor, rigidity, hypo-/bradykinesia, and/or postural impairment. Such patients should ideally be referred untreated to a MD specialist with expertise in the differential diagnosis of this condition [5], but practice and availability of MD services in some healthcare settings might limit this possibility. Therefore, knowledge of the present diagnostic criteria is necessary.

**Figure 1:**
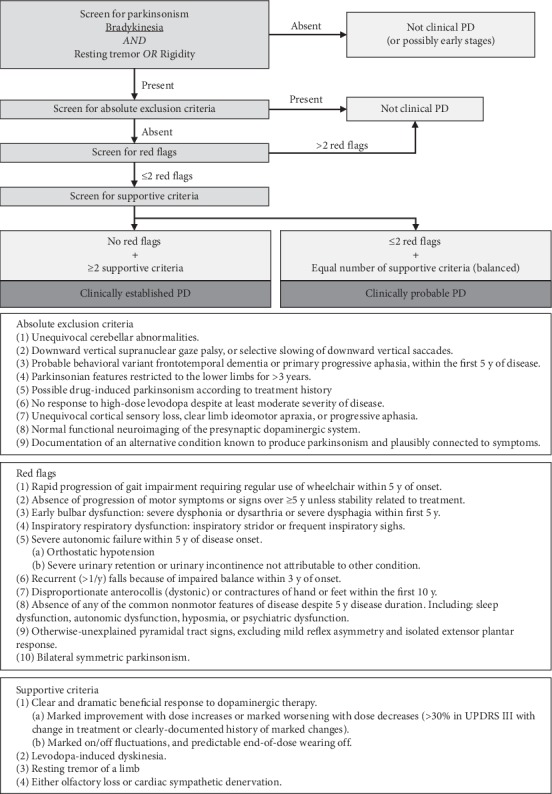
Summary diagram of Parkinson's disease diagnosis based on the diagnostic criteria of the movement disorder society [7].

Due to their relative specificity, only some of the clinical motor manifestations are taken into consideration as major criteria for the positive diagnosis of PD. The UK Brain Bank criteria are used in many centers for research, but also for diagnostic purposes (Supplementary table ([Supplementary-material supplementary-material-1])). This already traditional set of criteria has been confirmed by large neuropathological studies [6]. More recent diagnostic criteria are those of the International Parkinson and Movement Disorder Society (MDS, 2015). In the MDS set of criteria, the main criterion for diagnosis of PD is the presence of *parkinsonism*, defined as bradykinesia, in combination with either rigidity or resting tremor, or both [7]. All of these are motor manifestations. After confirmation of parkinsonism and evaluation of “absolute exclusion criteria” (symptoms not present in PD), “red flags” (symptoms atypical for PD), and also “supportive criteria” (symptoms typically or often present in PD), the specialist may diagnose either *clinically established PD* or *clinically probable PD*, as shown in Figure 1 [7]. The MDS criteria enable the diagnosis of PD at an earlier stage, take into account some early complex nonmotor aspects of the disease, and exclude posture and balance impairment as a major sign of the disease, as these are more related to the advanced stage.

Key messages for diagnosis of Parkinson's disease are as follows:

- There are two different diagnostic criteria for the clinical diagnosis of Parkinson's disease.
- The first and most important feature of Parkinson's disease is bradykinesia: tremor without bradykinesia is not enough for the diagnosis of parkinsonism or Parkinson's disease.
- Patients correctly diagnosed with Parkinson's disease should have a clear effect of their antiparkinsonian medications if the dose is sufficient. However, some motor symptoms such as tremor can result from a combination of dopaminergic and nondopaminergic brain lesions, which may contribute to variable response to levodopa across patients [8].

### 2.2. Heterogeneity of Symptoms in PD as a Progressive Disease

Typical MS include hypomimia, dysarthria, dysphagia, decreased arm swing, shuffling gait, festinations, freezing, difficulty rising from a chair, difficulties with turning in bed, cutting food, feeding and hygiene, micrographia, presence of inexhaustible glabellar reflex, blepharospasm or other dystonia, and camptocormia [9]. Some represent components of the natural history of the disease, while others are related to dopaminergic therapy. Additionally, NMS are more numerous than MS; the most frequent are presented in Table 1 [10–13]. Some NMS may occur as early as the premotor stage of PD (e.g., hyposmia, REM-sleep behavior disorder, depression, constipation, excessive daytime sleepiness, fatigue, pain, and erectile dysfunction), while others (dementia, hallucinations, etc.) usually occur later on in the course of the disease [13]. With all this in mind, there are several instruments that can aid the MD specialist in identification and assessment of severity of symptoms and disability level (Section 3).

### 2.3. Classifications of PD

All MS and NMS do not appear together in every patient. In fact, studies on larger cohorts reveal the existence of NMS + MS clusters in different clinical subtypes of PD [14–20]. Although there is no general consensus regarding PD subtypes, different variables taken into account in classifications (MS, NMS, genetic criteria, drug-induced complications, etc.) may point to some common conclusions. However, it is important to emphasize that there is no clear demarcation between phenotypic subtypes, as most of their clinical features overlap and only the dominance of some clusters of symptoms defines a certain subtype [21].

It is believed that the so-called “precision medicine” concept [22] will eventually guide the physician towards the best possible personalized care for an individual patient and his/her personal needs and requirements [21].

### 2.4. Complications Related to Pharmacological Treatment

The available pharmacological treatments for PD, based largely on dopaminergic drugs, are symptomatic only. They allow PD patients to improve their functional status and health-related quality of life (HRQoL), mainly during the early years after clinical onset of the disease. Unfortunately, presently available therapies do not halt disease progression. Biologic disease-related dysfunctions and the pharmacological properties of drugs interact and often induce drug-related complications such as motor and nonmotor fluctuations, dyskinesia, impulse control disorder (ICD), dopaminergic dysregulation syndrome (DDS), punding, dopamine-agonist withdrawal syndrome (DAWS), and levodopa withdrawal syndrome. Some complications can be ameliorated or delayed with optimization of oral therapy (Section 4.1) for several years until the advanced stage is reached.

*Levodopa-related clinical fluctuations* have various clinical presentations, and very often, the nonmotor fluctuations precede and/or accompany the motor ones [23]. Among the motor fluctuations, the earliest to occur is the “wearing-off” (end of dose deterioration), which is defined as a progressive shortening of the period between dose intakes of levodopa [10] due to a progressive shortening of the “on” time duration and an earlier than previously expected “off” occurrence. Other fluctuations include [10] suboptimal clinical response, delayed-on and no-on response (altered pharmacokinetics due to impaired motility of the upper gastrointestinal tract, mainly delayed emptying of the stomach), unpredictable “off” episodes, and freezing (motor blocks). However, it should be borne in mind that some fluctuations are not necessarily drug-related; for example, “on” freezing may be unresponsive to dopaminergic medication and may be present due to extensive lesions of the nondopaminergic structures of the brain.

The main clinical types of motor complications include (1) peak-dose dyskinesia, dominated by choreic movements and less frequently dystonic features during the “on” period, usually associated with high dopaminergic medication blood levels, (2) “off” dystonia, usually associated with low dopaminergic medication blood levels and “off” periods and that may manifest by painful foot inversion; off dystonia may develop as “early-morning dystonia” that manifests on awakening before the first dose of levodopa), daytime and nocturnal dystonia; and, (3) diphasic (biphasic) dyskinesia that usually manifests as choreic and/or dystonic movements at the beginning and the end of the “on” period. Dyskinesia represents an abnormal hyperkinetic movement that is different to those specific to the natural evolution of PD. It is related to the effects of the dopaminergic therapy (mainly levodopa) in interaction with the neuroplasticity mechanisms. Dyskinesia may impair daily activities significantly (troublesome dyskinesia) or nonsignificantly (nontroublesome dyskinesia) [24]. This clinical distinction of severity is important for treatment decisions.

ICDs include pathological gambling, compulsive buying, hypersexual disorders, or binge eating, and ICD-related disorders such as DDS, punding, compulsive hoarding, or aimless wandering [25]. These disorders have been associated to various drugs, including levodopa, amantadine, and rasagiline. Intake of dopamine agonists has been reported as a major risk factor for developing ICDs [25, 26], but it remains unclear whether long-acting agonists and nonoral formulations may reduce the risk [27], nor association with DA dose, treatment duration, or peak dose has been established [25, 26]. At any rate, prevalence of ICDs increases over time in PD. It has been described that early onset of PD and presence of motor complications may be associated to higher risk of ICDs [25, 26].

It should be borne in mind that not all clinical manifestations of PD are dopaminergic in nature and that nondopaminergic symptoms (such as autonomic dysfunction, sleep disorders, pain syndromes, mood disturbances, and dementia) are largely unresponsive to currently available therapeutic possibilities. These nondopaminergic symptoms often dominate in advanced stages of the disease and cause more severe disability and impairment of quality of life (QoL) than dopaminergic symptoms [28].

### 2.5. Progressing to Advanced Disease

As PD is clinically very heterogenous and progressive in nature, it is not an easy task to define the concept of advanced PD. Various efforts in the form of systematic analyses, expert consensuses from national steering committees in Europe [24], and Delphi studies with specialized MD panelists [29, 30] have been made to propose criteria. Advanced PD is generally reached when patients develop characteristic complications associated with long-term levodopa treatment, uncontrolled with optimized conventional therapies [29]. The common feature in all proposals to characterize advanced PD is the impact of disease manifestations on disability and QoL. However the concept is “still controversial and is variably applied to patients with long disease duration or with motor fluctuations and severe or moderate dyskinesia, with impairment of gait, equilibrium, cognition or neuropsychiatric symptoms” [29]. The authors of a recently published consensus [29] found the development of severe motor fluctuations with disabling “off” periods to be the most important factor; both MS and NMS, either related to the evolution of the disease or to the long-term levodopa therapy, are considered essential for the diagnosis of advanced PD. Accordingly, there is growing awareness of nonmotor aspects of PD as indicators of the advancing course. NMS, unfortunately, are not properly reflected in the usual scale-based assessments, which are largely focused on MS [31]. Practical cues on when advanced disease might be suspected are described in Section 4.3.

Key messages for follow-up in Parkinson's disease are as follows:

- Patient diaries may be used to evaluate ON time without dyskinesia, ON time with dyskinesia and OFF time once motor fluctuations appear. However, appropriate training is required for reliable keeping of the diaries (Supplementary file).
- Nonmotor symptoms and potential side effects of medications are often not reported by the patients. Both aspects should be actively screened by the treating neurologist.

## 3. Evaluation of Clinical Manifestations

Numerous rating scales have been developed to assess multidimensional aspects of PD more reliably. A detailed description of diagnostic tools is beyond the scope of this article, but GNs should be familiar with updated information on the various instruments used in the MD specialist clinic and their interpretation. The most relevant tools evaluating MS and NMS, dyskinesia, sleep quality, and HRQoL are summarized and described in Table 2, along with the most important grading schemes. Knowledge of common scales and their interpretation may be of use for the GN when communicating with an MD specialist and discussing results. When interpreting results, the *minimal clinically important difference* (MCID) threshold values may also be considered. MCID is the smallest change in an outcome measure that a patient or clinician would identify as important, therefore representing the threshold above which the outcome is experienced as relevant by the patient. This parameter may be more useful to the MD specialist than statistical significance, since some changes may be statistically significant in the research literature but reflect no clinical relevance.

## 4. Management according to the Disease Stage

Several studies support the early introduction of antiparkinsonian treatment as soon as the diagnosis is confirmed [52]. Oral levodopa, the initial gold-standard therapy for PD, is still the most effective and widely used therapeutic option in the treatment of this neurodegenerative disorder. However, its use eventually results in the development of motor fluctuations and levodopa-induced dyskinesia (LID) [53]. Nearly 40% of PD patients develop LID after 4 to 6 years of levodopa treatment [54].

Therefore, particularly in younger patients in whom motor complications typically occur earlier and are more severe, pharmacological treatment should be started with MAO B inhibitors or dopamine agonists, adding levodopa later on as soon as needed. One of the mechanisms by which motor fluctuations and LID occur is the intermittent, nonphysiological pulsatile manner in which oral levodopa stimulates dopaminergic receptors [55, 56]. When symptoms cannot be optimally alleviated with conventional oral medications, it is time to apply the strategies of continuous dopaminergic administration by introducing the so-called “advanced therapies” or “device-aided treatments” (Section 4.2). However, before reaching this advanced stage, the appropriate use of available oral treatments might help optimize conventional pharmacotherapy in patients (Section 4.1), and some nonpharmacological approaches should be attempted in PD management (Section 4.3).

### 4.1. Optimization of Conventional Oral Medication

In the early stages of PD, symptoms can generally be alleviated or abolished by MAO-B inhibitors, dopamine agonists, or with low-dose levodopa taken three or four times daily (“honeymoon period”). Initial choice likely has no long term effect [57], so levodopa treatment in younger patients who need more powerful motor improvement should not be avoided. In fact, most of the patients who started with dopamine agonists will require levodopa treatment after several years. In addition, as previously mentioned, patients with early onset of PD are more prone to develop ICD (more often caused by dopamine agonists). In summary, treatment should be considered carefully and individually based on personality and psychiatric and physical comorbidities.

As the disease progresses, successful management will increasingly require addition of more antiparkinsonian medications, as well as increased dosages and frequency of drug intake. A detailed history should be taken from the patient and his/her caregiver to identify problems, such as underdosing, new motor and nonmotor signs, motor and nonmotor fluctuations, and motor and nonmotor side effects of medication [58]. While proper identification of these issues is crucial for further management, there is no definitive expert consensus on the actual *medication picture* of a candidate for switching to advanced PD therapies; that is, there is no agreement on the combination of drugs and dosages that would help define a patient no longer well controlled with regular oral medication. Nevertheless, there are several good practice points and generally accepted interventions for troublesome issues, such as morning “off,” wearing “off,” delayed and failed “on,” peak-of-dose dyskinesia, biphasic dyskinesia, troublesome night-time “off” periods, and others (Table 3), requiring assessment by an MD specialist earlier in the disease course.

For practical purposes, patients should be using ≥5 effective doses of levodopa daily before considering transition to advanced therapies [30]. If “off” periods persist, adding MAO-B inhibitors and/or COMT-inhibitors should be considered [59]. If possible, dopamine agonists should be used at the maximum tolerated dose (not inducing side-effects) and amantadine should be considered for severe dyskinesia [61]. Motor fluctuations and dyskinesias are not necessarily associated with the duration of levodopa therapy, but rather with longer disease duration and higher levodopa daily dose [62]. If the effect of individual levodopa doses is shorter than 3 hours, it is unlikely that further shortening of intervals will lead to sufficient control of symptoms [63].

Presently, there is no consensual agreement among MD specialists as to the defining clinical features of advanced disease [64] and when advanced therapies should be introduced. The GN should be familiar with signs and features that lead to suspicion of advanced PD and contact an MD specialist in a timely manner, so an appropriate assessment and complementary interventions can be carried out. The disease duration and the emergence of motor [65] and nonmotor [66] complications are crucial for the diagnosis of advanced PD. Antonini et al. [30] published a series of indicators for transition to advanced PD achieved with a Delphi approach that may provide good orientation (Table 4).

Key messages for treatment of Parkinson's disease (initial stages) are as follows:

- When choosing medication, individual approach is required based on symptoms and preference.
- Start low and go slow until reaching good clinical benefit
- Medications with a more continuous stimulation profile, such as dopamine agonists or MAO-B inhibitors, are preferred in initial stages if appropriate for the clinical profile of the patient
- Levodopa should not be avoided at all costs, even in initial stages. Consider levodopa if other medications are not indicated or not effective.

### 4.2. Advanced Therapies for Advanced Stages

In patients in whom conventional oral pharmacotherapy has been exhausted and cannot be optimized, three main device-assisted therapies should be considered, all of them based on the concept of CDS: (1) continuous subcutaneous infusion of apomorphine; (2) intra-intestinal infusion of levodopa-carbidopa gel (LCIG); and (3) deep-brain stimulation (DBS). The optimal timing for initiating these advanced therapies to improve QoL and prevent complications is critical and requires that patients and caregivers be informed early about the evolution to later stages of the disease with their complications [21]. As the decision to initiate any of these therapies should be made by a MD specialist and a multidisciplinary team [69], the role of the GN in timely referral is critical for adequate patient management. Currently, there are no large studies comparing the procedures [70], and the choice between therapies is based upon many considerations, as shown in Table 4.

*Apomorphine*, a D1 and D2 dopamine receptor agonist, has rapid onset of action and is used in earlier stages as rescue injections during “off” periods. In advanced PD, it is delivered as a continuous subcutaneous infusion by means of a portable pump. It has shown effectiveness in treating MS and some NMS in advanced stages of the disease [71, 72].

*LCIG* is delivered directly to the proximal jejunum via a percutaneous gastrojejunostomy (PEG-J) tube connected to a portable infusion pump [73]. This therapy is used to avoid erratic gastric emptying and to improve intestinal absorption. LCIG is an effective treatment for reducing motor fluctuations, improving “on” time without dyskinesia and improving HRQoL in advanced PD [74].

*Deep brain stimulation (DBS)* is a functional neurosurgical procedure that can be used to treat motor fluctuations, dyskinesia, and tremor [75]. It has also been shown to improve HRQoL [76]. However, levodopa-unresponsive symptoms in advanced PD (e.g., gait instability, psychiatric disorders, cognitive impairment, dysarthria, and autonomic dysfunction) are unlikely to improve with DBS [77].

The key to the success of any advanced therapy is appropriate patient selection, including the patient's preference whenever possible. A multidisciplinary team should be involved in patient and treatment selection, and its participation is also imperative in the follow-up [69].

Key messages for treatment of Parkinson's disease (advanced stages) are as follows:

- If good ON period is shorter than 3 hours, further adjustments of oral medications will most likely fail, and advanced treatments should be considered.
- Indication of advanced treatment options *should not be* delayed if standard medications fail to control sufficiently motor fluctuations.
- *Do not delay* referral *or contact* with a team specialized in movement disorders if advanced disease is suspected.

### 4.3. Nonpharmacological Interventions across All Stages

The main goal of any management should be to maintain acceptable levels of functioning and independence. In advanced PD, this can be achieved with a careful combination of drugs and supportive nonpharmacological therapy [78] in the context of collaboration between the GN, the MD specialist, and the multidisciplinary team. Supportive nonpharmacological management in advanced PD patients should include physical rehabilitation, psychological support, occupational therapy, speech, language and swallowing therapy, and nutrition [78, 79], among other possible interventions (Table 5). Balance and gait have been shown to improve with physical therapy and exercise, thus reducing the risk of falling [86, 87]. Physical activity has beneficial effects in PD, and forms that have shown benefit include aerobic exercise including dancing and treadmill training, resistance training, and traditional activities such as Tai Chi and yoga [88]. High intensity training may also improve the motor symptoms and limitations [89]. Speech therapy has been successfully used to improve hypophonic and hypokinetic speech and related functions, such as swallowing problems associated with PD [82, 90]. Integration of medical and nonmedical treatments is most efficiently planned by members of a multidisciplinary team, usually established as part of tertiary MD centers.

## 5. Multidisciplinary and Team Approach across All Stages

When a broad range of MS and NMS is set within the framework of individual requirements and priorities of each patient, the need for close and personalized care becomes obvious. This is best achieved by a multidisciplinary team. Such a team is often part of a tertiary center for movement disorders. From a practical point of view, the GN should find out whether there is a multidisciplinary team in the local hospital or conveniently located elsewhere for the patient. Contact with a reference team may be of use as early as the initial stages of the disease to provide advice or support to resolve common issues. However, members of a multidisciplinary team will play an essential role, particularly in advanced stages. The GN should contact the team coordinator (often a specialized PD nurse) to help as a pivotal point of communication with other members of the team. Cooperation between the GN, caregiver, patient, and the multidisciplinary team is a multichannel and bidirectional pathway and, from time to time, it may be useful for the GN (and/or patient and caregiver) to attend a team meeting.

At these meetings, the GN will establish contact with the core members of the team, particularly the neurologist-MD specialist, PD nurse, and—when and if needed—also with a psychologist, social worker, physiotherapist, occupational therapist, and speech therapist. These professionals are specially trained to provide advice, education, and support, which should be tailored to the needs of the individual patient and can be obtained much faster when in direct communication with experts. If needed, other professionals such as a sex therapist, dietitian, psychiatrist, gastroenterologist, and neurosurgeon might be involved, usually through the team meetings. Table 5 lists the activities and kind of support the usual multidisciplinary team members may provide.

## 6. Conclusion

The progressive and multifocal nature of PD adds complexity to the management of this disease, with important and increasing prevalence in the aging population. Recent advances in the knowledge of PD provide growing insight not only into mechanisms of the disorder but also into greater understanding of patients' needs and the use of relevant tools to improve their QoL. General neurologists attending PD patients at any stage may benefit from a practical update of this knowledge. There is a lot that GNs can do for their PD patients in earlier stages, as well as in advanced stages, particularly when in good and timely communication with a multidisciplinary team, whether for advice, support interventions, or referral when necessary.

## Figures and Tables

**Table 1:** Most frequently described nonmotor symptoms of Parkinson's disease due to natural history of the disease or related to therapy [10–13].

Domain	Symptoms
Autonomic	Blood pressure variations with orthostatic hypotension, tachycardia, urinary disturbances (such as urgency, frequency), nocturia, sexual dysfunction, hypersexuality (likely to be drug-induced), paroxysmal sweating, seborrhea, xerostomia (“dry eyes”), facial hyperemia, mydriasis, pallor
Gastrointestinal (partly related to dysautonomia)	Drooling of saliva, ageusia, dysphagia, constipation, fecal incontinence, eructation, meteorism
Sleep	REM sleep behavior disorder (RBD), excessive daytime sleepiness, vivid dreams, insomnia, periodic limb movements (PLM), restless legs syndrome (RLS)
Neuropsychiatric	Cognitive impairment (including mild cognitive impairment and dementia), depression, anhedonia, apathy, anxiety, panic attacks, delirium, hallucinations, illusions, delusions, impulse control disorder (ICD), dopaminergic dysregulation syndrome, dopamine agonist withdrawal syndrome (DAWS)
Sensory	Pain, olfactory disturbance, blurred vision, visual discrimination deficits (also related to neurocognitive impairment)
Miscellaneous	Fatigue, diplopia, weight loss or weight gain (often drug- and evolution-related)

Note: this list is not exhaustive. Abbreviations are given for terms that are often used in the abbreviated form.

**Table 2:** Instruments for general assessment, health-related quality of life assessment, and complications of Parkinson's disease.

Assessment tool	Abbrev.	Measures	Grading severity				MCID			Quick tips
Hoehn and Yahr Scale [32]	HYS	General assessment	Mild: HYS 1 and 2 Moderate: HYS 3 Severe: HYS 4 and 5							Besides the original version, a modified version (mHYS) is also available

Unified Parkinson's Disease Rating Scale [33]	UPDRS	General assessment, 4 subscales: Part I: mentation, behavior and mood Part II: activities of daily living Part III: motor examination Part IV: treatment complications					Part II: 0.7 (early PD) [34], 2 (HYS 1&2) [35], 3 points (HYS 2.5–3) [35] Part III: 2.4 (early PD) [34], 5 (HYS 1–3) [35], 3.5 (advanced) [36]			In most cases, the total score is calculated as the sum of parts I + II + III

Movement Disorders Society-Sponsored Unified Parkinson's Disease Rating Scale [37]	MDS-UPDRS	General assessment, 4 subscales: Part I: non-motor experiences of daily living Part II: motor experiences of daily living Part III: motor examination Part IV: motor complications	Score [38]:				Score change:			Solves several ambiguities of UPDRS; subscales should be interpreted separately
				Mild	Moderate	Severe		Improvement	Worsening	
			Part I	≤10	11–21	≥22	Part I [39]	−2.64	2.45	
			Part II	≤12	13–29	≥30	Part II [39]	−3.05	2.51	
			Part III	≤32	33–58	≥59	Part III [40]	−3.25	4.63	

Parkinson's Disease Questionnaire (39 items) [41]	PDQ-39	Disease-specific health-related quality of life 39 items grouped into 8 domains making up a summary index					Score change [42]: Improvement −4.72 points, Worsening 4.22 points			One of the most relevant instruments

Parkinson's Disease Questionnaire (8 items) [43]	PDQ-8	Disease-specific health-related quality of life. 8 questions making up a summary index					Improvement −5.94 [42] (range: −4.6, −10) [36, 44] Worsening 4.91 points [42]			This is the “short” version of PDQ-39

Nonmotor Symptoms Scale [45]	NMSS	Measures nine domains: Cardiovascular, sleep/fatigue, mood/cognition, perception problems, attention & memory, gastrointestinal- urinary, sexual function, and miscellaneous	Absent or mild: 0–20 points Moderate: 21–40 points Severe: 41–70 points Very severe: ≥ 71 points [46]							There is also a screening instrument (NMSQ); NMSS assesses severity and frequency

Parkinson's Disease Sleep Scale 2nd version [47]	PDSS-2	Overall sleep quality; 3 subscales on motor problems at night, PD problems at night and disturbed sleep	Absent or minimal: 0–10 points Mild-moderate: 11–20 points Severe: ≥20 points [48]				Improvement −3.44 points Worsening 2.07 points [49]			PDSS-2 is an improved version of the original PDSS
Unified Dyskinesia Rating Scale [50]	UDysRS	Overall assessment of dyskinesia: Part I: historical On-Dyskinesia Part II: historical Off-Dystonia Part III: objective Impairment Part IV: objective disability					Part III: Improvement 2.32 points Worsening 2.76 points [51]			UDysRS is the most comprehensive dyskinesia scale

MCID, minimal clinically important difference; PD, Parkinson's disease.

**Table 3:** Pragmatic optimization of oral medications for selected issues related to Parkinson's disease management.

A. Most relevant motor and non-motor symptoms that may lead to suspicion of inadequately controlled Parkinson's disease (ranked by level of importance) [58]		
*Motor*	*Non-motor*	

(1) troublesome level of motor fluctuations (2) two hours of the day with “off” symptoms (3) At least 1 hour of the day with troublesome dyskinesia (4) Presence of motor complications (5) daily multiple oral levodopa doses)	(1a) Troublesome hallucinations/psychosis (1b) Non-motor symptom fluctuations (2a) Impulse control disorder (2b) Troublesome level of nighttime sleep disturbances (3a) Troublesome level of depression (3b) Troublesome level of daytime sleepiness	

B. Pragmatic approach: questions for the patient	If answer is NO: potential problem	Potential solutions

Are your symptoms sufficiently controlled?	Insufficient effect–the patient might be underdosed	(i) Increase dose of dopamine agonist (ii) Increase individual levodopa doses
When you wake up in the morning, is your mobility acceptable? If not, how long does it take for your medication to start working?	Troublesome morning “off” time	(i) Prescribe morning levodopa for immediately after waking up (ii) Increase dopamine agonist dose (use more in the evening) (iii) If morning levodopa dose is less effective compared to other doses–increase this individual dose
When your medication starts working, does the effect last until the next dose? If not, how long do you experience symptoms?	Wearing off (motor or non-motor symptoms)	(i) Increase dopamine agonist dose (ii) Prescribe more frequent doses of levodopa (iii) Add a COMT inhibitor (e.g. entacapone, tolcapone) [59, 60] (iv) Add an MAO-B inhibitor (e.g. selegiline, rasagiline [59, 60]
Does the effect of some of your doses take long to start or do you completely fail to experience its effects?	Delayed “on” Failed “on”	(i) Indicate the use of levodopa always at least 30–45 minutes before or after meals (not with food) (ii) Prescribe prokinetics (e.g. domperidone) (iii) Exclude *Helicobacter pylori* and/or SIBO syndrome
Do you have excessive involuntary movements when your medication is working?	Dyskinesia	(i) Prescribe levodopa in lower doses and more frequently (ii) Add amantadine [61]
Is your mobility during the night acceptable?	Troublesome nighttime “off”	(i) Prescribe immediate-release levodopa for nighttime wake-ups (ii) Increase dopamine agonist (to use more in the evening)

COMT, catechol-O-methyl transferase; MAO-B, monoamine oxidase B; SIBO, small intestine bacterial overgrowth.

**Table 4:** Characteristics of patients with Parkinson's disease who might be eligible for advanced device-aided therapies [29, 30, 58].

Characteristics of patients as proposed by expert-opinion studies			
Motor	Non-motor	Function	

(i) Troublesome level of motor fluctuations [29, 30, 58] (ii) At least 1 hour of the day with troublesome dyskinesia [30, 58] (iii) At least 2 hours of the day with “off” time [29, 30, 58] (iv) Off-period postural instability [29, 30] (v) Dystonia with pain [30] (vi) Freezing of gait during “off” time [30] (vii) Daily multiple oral levodopa doses [58]	(i) Non-motor symptom fluctuations [58] (ii) Impulse control disorder [58] Troublesome level of nighttime sleep disturbances [30, 58] (iii) Severe dysphagia and recurrent falls [29] (iv) Troublesome level of daytime sleepiness [58] (v) Troublesome level of anxiety [58]	(i) Needing help with activities of daily living at least some of the time (limited) [29, 30]	

Proposed profiles according to clinical characteristics [30]			
Characteristics	Apomorphine	Deep brain stimulation (DBS)	Levodopa carbidopa Intestinal gel (LCIG)

Younger age (<70 years)	Probably good candidate [58]	*Probably good candidate* [58]	*Probably good candidate* [58]
Older age (>70 years)	*Probably good candidate* [67, 68]	Possible candidate [67, 68]	*Probably good candidate* [67, 68]
Good levodopa response	Probably good candidate [58]	Probably good candidate [58]	Definitely good candidate [58]
Levodopa-resistant tremor	Not a candidate [58, 67]	*Definitely good candidate* [58, 67]	Not a candidate [58, 67]
Troublesome dyskinesia	Possible candidate	*Probably good candidate* [58]	*Probably good candidate* [58]
Good cognitive function	*Probably good candidate* [58]	*Probably good candidate* [58]	*Probably good candidate* [58]
Nighttime sleep disturbances	Possible candidate [58]	Possible candidate [58]	Possible candidate [58]
Pain	Possible candidate [58]	Possible candidate [58]	Possible candidate [58]
Impulse control disorder	Not a candidate [58, 67, 68]	Possible candidate [58, 67, 68]	Possible candidate [58, 67, 68]
Depression	Possible candidate [58, 68]	Not a candidate [58, 68]	Possible candidate [58, 68]
Apathy	Possible candidate [58]	Not a candidate [58]	Possible candidate [58]
Anxiety	Not a candidate [58]	Possible candidate [58]	Possible candidate [58]
Mild dementia	Possible candidate [67, 68]	Not a candidate [67, 68]	Possible candidate [67, 68]
Multimorbidity	Possible candidate [67]/Not a candidate [68]	Not a candidate [67]	Possible candidate [67]/Not a candidate [68]
Lack of social support/caregiver	Not a candidate [67]	Possible candidate [67]	Not a candidate [67]
Excessive daytime sleepiness	Not a candidate [68]	Possible candidate [68]	Possible candidate [68]
Dysphagia	*Probably good candidate* [68]	Not a candidate [68]	*Probably good candidate* [68]

In all studies cited, the recommendations are based on clinical experience and expert opinion in the absence of robust comparative evidence. If “possible” or “probably good” candidate is described, check warnings for use in the label that should be taken into consideration.

**Table 5:** Some key members of a Movement Disorders Team, and the activities they may perform as non-pharmacological support for the patient and the attending neurologist.

Specialized nurse [80]	(i) Nurse specialized in Parkinson's Disease (ii) Often central coordination role in multidisciplinary team (iii) Usually in closest contact with the patient and his family (iv) Participates in clinical monitoring and adjustment of medicines (v) May be a reliable source of information about clinical and social matters of concern to the attending neurologist

Physiotherapist [81]	(i) Uses a range of techniques and strategies to help in maintaining good posture, balance and fitness through exercise (ii) Trains the patient in external cueing for improving gait and balance, helping to prevent falls: Individual rehabilitation session 2–3 times weekly may reduce the risk of falls (iii) Trains the patient for maintaining effective breathing and helping with pain relief

Speech therapist	(i) Should be involved as soon as the patient starts experiencing difficulties with communication and/or swallowing (ii) May provide intensive and efficient interventions, e.g.: (1) Lee silverman voice treatment [82, 83], shown to reduce hypophonia & hypokinetic dysarthria (2) Training for expiratory muscle strength to reduce incidence of aspiration [84] (3) Techniques for improving swallowing and facial expression

Occupational therapist [85]	(i) Should be consulted on aspects of daily living, such as finding ways to continue working, keeping up with hobbies and leisure interests. These interventions may improve functional activities (ii) May help with creating safer environments or providing equipment to maintain independence

Psychologist	(i) Participates in the diagnostic set-up (ii) Key for advising patients and caregivers about psychological problems and how to cope with the emotional burden of the disease

Social worker	(i) Assesses social conditions and helps with psychosocial adjustments (ii) Instrumental in educational interventions for the patient and caregivers (iii) Provides support and information when the patient needs to be transferred to a nursing home

## Supplementary Materials

Supplementary Materials The supplementary material of this article consists of a table reproducing the United Kingdom Parkinson's Disease Society Brain Bank clinical diagnostic criteria (as published in 1992) and an example of a patient motor diary.
