# Supplementary material for: Update on the Management of Parkinson's Disease for General Neurologists
Source: Parkinsons Dis. 2020 Mar 26;2020:9131474. doi: 10.1155/2020/9131474 (PMC7136815; doi:10.1155/2020/9131474)
Supplement: Supplementary Materials — The supplementary material of this article consists of a table reproducing the United Kingdom Parkinson's Disease Society Brain Bank clinical diagnostic criteria (as published in 1992) and an example of a patient motor diary. [file 9131474.f1.docx]

| **Supplementary table.** The United Kingdom Parkinson’s Disease Society Brain Bank clinical diagnostic criteria |
| --- |
| **Step 1. Diagnosis of Parkinsonian Syndrome** |
| - Bradykinesia   And at least one of the following:   - Muscular rigidity - 4-6-Hz rest tremor - Postural instability not caused by primary visual, vestibular, cerebellar, or proprioceptive dysfunction |
| **Step 2. Exclusion criteria for Parkinson’s disease** |
| - History of repeated strokes with stepwise progression of parkinsonian features - History of repeated head injury, history of definite encephalitis - Oculogyric crises - Neuroleptic treatment at onset of symptoms - More than one affected relative - Sustained remission - Strictly unilateral features after 3 years - Supranuclear gaze palsy - Cerebellar signs - Early severe autonomic involvement - Early severe dementia with disturbances of memory, language, and praxis - Babinski sign - Presence of cerebral tumour or communication hydrocephalus on imaging study - Negative response to large doses of levodopa in absence of malabsorption - MPTP exposure |
| **Step 3. Supportive prospective positive criteria for Parkinson’s disease** |
| ≥3 required for diagnosis of definite PD in combination with Step 1   - Unilateral onset - Rest tremor present - Progressive disorder - Persistent asymmetry affecting side of onset most - Excellent response (70-100%) to levodopa - Severe levodopa-induced chorea - Levodopa response for ≥5 years - Clinical course of ≥10 years |
| MPTP, 1-methyl-4-phenyl-1,2,3,6-tetrahydropyridine. Reproduced from Hughes AJ, et al. JNNP 1992;55:181-184. |

**EXAMPLE Patient Motor Diary**

## Instructions

Please complete your 24-hour diary pertaining to your Parkinson’s symptoms before your next visit. Your cooperation be very helpful to your neurologist to finely tune your medical management.

This diary is divided into 30-minute sections. Each day starts at midnight (first column) and ends at 11:30 p. m. at the bottom of the second column. Please record how you would rate your mobility for each 30-minute period by marking with an X the corresponding box in each line; also mark when you were asleep. Remember that you should only mark one answer for each half hour period. Although you may have several mobility issues within a 30-minute period, record your status for the MAJORITY of that period.

## Definitions

| ON | Good or practically normal mobility. |
| --- | --- |
| ON with Troublesome Dyskinesia | Troubled by involuntary twisting, turning movements. These movements are different from the rhythmic “tremor” which is a symptom of Parkinson’s Disease itself. |
| OFF | Stiffness, marked decrease in mobility, or immobility. |
| ASLEEP | Time spent asleep. |

# Diary Date: Day / Month /Year

| Monday | Tuesday | Wednesday | Thursday | Friday | Saturday | Sunday |
| --- | --- | --- | --- | --- | --- | --- |

| **Time from**  **MIDNIGHT**  **(AM)** | | **ON** | **ON with**  **Troublesome**  **Dyskinesia** | **OFF** | **ASLEEP** | **Time from NOON**  **(PM)** | | **ON** | **ON with**  **Troublesome**  **Dyskinesia** | **OFF** | **ASLEEP** |
| --- | --- | --- | --- | --- | --- | --- | --- | --- | --- | --- | --- |
| **12:00 AM** | **12:30 AM** | □ | □ | □ | □ | **12:00 PM** | **12:30 PM** | □ | □ | □ | □ |
| **12:30 AM** | **1:00 AM** | □ | □ | □ | □ | **12:30 PM** | **1:00 PM** | □ | □ | □ | □ |
| **1:00 AM** | **1:30 AM** | □ | □ | □ | □ | **1:00 PM** | **1:30 PM** | □ | □ | □ | □ |
| **1:30:00** | **2:00 AM** | □ | □ | □ | □ | **1:30:00** | **2:00 PM** | □ | □ | □ | □ |
| **2:00 AM** | **2:30 AM** | □ | □ | □ | □ | **2:00 PM** | **2:30 PM** | □ | □ | □ | □ |
| **2:30 AM** | **3:00 AM** | □ | □ | □ | □ | **2:30 PM** | **3:00 PM** | □ | □ | □ | □ |
| **3:00 AM** | **3:30 AM** | □ | □ | □ | □ | **3:00 PM** | **3:30 PM** | □ | □ | □ | □ |
| **3:30 AM** | **4:00 AM** | □ | □ | □ | □ | **3:30 PM** | **4:00 PM** | □ | □ | □ | □ |
| **4:00 AM** | **4:30 AM** | □ | □ | □ | □ | **4:00 PM** | **4:30 PM** | □ | □ | □ | □ |
| **4:30 AM** | **5:00 AM** | □ | □ | □ | □ | **4:30 PM** | **5:00 PM** | □ | □ | □ | □ |
| **5:00 AM** | **5:30 AM** | □ | □ | □ | □ | **5:00 PM** | **5:30 PM** | □ | □ | □ | □ |
| **5:30 AM** | **6:00 AM** | □ | □ | □ | □ | **5:30 PM** | **6:00 PM** | □ | □ | □ | □ |
| **6:00 AM** | **6:30 AM** | □ | □ | □ | □ | **6:00 PM** | **6:30 PM** | □ | □ | □ | □ |
| **6:30 AM** | **7:00 AM** | □ | □ | □ | □ | **6:30 PM** | **7:00 PM** | □ | □ | □ | □ |
| **7:00 AM** | **7:30 AM** | □ | □ | □ | □ | **7:00 PM** | **7:30 PM** | □ | □ | □ | □ |
| **7:30 AM** | **8:00 AM** | □ | □ | □ | □ | **7:30 PM** | **8:00 PM** | □ | □ | □ | □ |
| **8:00 AM** | **8:30 AM** | □ | □ | □ | □ | **8:00 PM** | **8:30 PM** | □ | □ | □ | □ |
| **8:30 AM** | **9:00 AM** | □ | □ | □ | □ | **8:30 PM** | **9:00 PM** | □ | □ | □ | □ |
| **9:00 AM** | **9:30 AM** | □ | □ | □ | □ | **9:00 PM** | **9:30 PM** | □ | □ | □ | □ |
| **9:30 AM** | **10:00 AM** | □ | □ | □ | □ | **9:30 PM** | **10:00 PM** | □ | □ | □ | □ |
| **10:00 AM** | **10:30 AM** | □ | □ | □ | □ | **10:00 PM** | **10:30 PM** | □ | □ | □ | □ |
| **10:30 AM** | **11:00 AM** | □ | □ | □ | □ | **10:30 PM** | **11:00 PM** | □ | □ | □ | □ |
| **11:00 AM** | **11:30 AM** | □ | □ | □ | □ | **11:00 PM** | **11:30 PM** | □ | □ | □ | □ |
| **11:30 AM** | **12:00 PM** | □ | □ | □ | □ | **11:30 PM** | **12:00 AM** | □ | □ | □ | □ |
